# Advance in Nrf2 Signaling Pathway in Leishmaniasis

**DOI:** 10.3390/biomedicines12112525

**Published:** 2024-11-04

**Authors:** Sarmistha Saha, Nadezhda Sachivkina, Olga Kuznetsova, Ekaterina Neborak, Natallia Zhabo

**Affiliations:** 1 Department of Biotechnology, Institute of Applied Sciences & Humanities, GLA University, Mathura 281406, Uttar Pradesh, India; 2 Department of Microbiology V.S. Kiktenko, Institute of Medicine, Peoples’ Friendship University of Russia (RUDN University), 117198 Moscow, Russia; sachivkina@yandex.ru; 3 Department of Biochemistry T.T. Berezov, Institute of Medicine, Peoples’ Friendship University of Russia (RUDN University), 117198 Moscow, Russia; olya.k@mail.ru (O.K.); neborak_ev@pfur.ru (E.N.); 4 Department of Foreign Languages, Institute of Medicine, Peoples’ Friendship University of Russia (RUDN University), 117198 Moscow, Russia; lys11@yandex.ru

**Keywords:** *Leishmania*, macrophage, HO-1, NRF2, gene expression

## Abstract

One of the main components of innate defense against invasive parasites is oxidative stress, which is brought on by reactive oxygen species (ROS). On the other hand, oxidative stressors serve two purposes: free radicals aid in the elimination of pathogens, but they can also set off inflammation, which leads to tissue damage. Nuclear factor erythroid 2-related factor 2 (Nrf2) is a transcription factor that controls the expression of numerous genes involved in the body’s defense against oxidative stress brought on by aging, inflammation, tissue damage, and other pathological consequences. From cutaneous to visceral forms, *Leishmania* parasites invade macrophages and cause a wide range of human pathologies. *Leishmania* parasites have a wide range of adaptive mechanisms that disrupt several macrophage functions by altering host signaling pathways. An increasing amount of data are corroborating the idea that one of the primary antioxidant routes to counteract this oxidative burst against parasites is NRF2 signaling, which also interferes with immune responses. The nature and potency of the host immune response, as well as interactions between the invading *Leishmania* spp., will ascertain the course of infection and the parasites’ eventual survival or eradication. The molecular processes via which Nrf2 coordinates such intricate networks comprising various pathways remain to be completely understood. In light of NRF2’s significant contribution to oxidative stress, we examine the NRF2 antioxidant pathway’s activation mechanism in *Leishmania* infection in this review. Thus, this review will examine the relationship between Nrf2 signaling and leishmaniasis, as well as explore potential therapeutic strategies for modifying this system.

## 1. Introduction

A wide range of diseases, particularly in developing nations, can be brought on by parasitic organisms. The initial non-specific line of protection against parasites is innate immunity. Due to their host-related reactive oxygen species (ROS), many parasites are more vulnerable. Thus, one of the primary foundations of innate immunity is the inflammatory response against parasitic microbes, which is accompanied by the production of ROS. An imbalance between oxidants and antioxidants brought on by unchecked inflammation may seriously harm the host [1,2]. Increasing antioxidant expression may be able to stop oxidant-mediated cytotoxicity and apoptosis in the host cells in different infections [3,4].

Globally distributed, human cutaneous leishmaniasis (CL) is thought to affect between 0.7 and 1.2 million people annually [5]. *Leishmania* parasites have a wide range of adaptive mechanisms that disrupt several macrophage functions by altering host signaling pathways [6]. Humans experience varying clinical symptoms as a result of infection by different species of *Leishmania* and the immunological response. It has been known for some time that certain *Leishmania* species include the *Leishmania* RNA virus (LRV), a viral endosymbiont, in their cytoplasm and as a member of the Totiviridae family, LRV is distinguished by icosahedral particles found in a variety of protozoa, such as *Toxoplasma gondi*, *Entamoeba*, and *Trichomonas vaginalis* [7]. The non-segmented double-stranded RNA genome of the virus, which is responsible for the replication of the dsRNA virus, encodes a capsid protein and a capsid-RNA-dependent RNA polymerase fusion protein. The viral particles have a diameter of 30–40 nm. Since the two *Leishmania* subgenera have different LRV sequences, they have been classified as LRV1 and LRV2, respectively, in *L. Viannia* and *L. Leishmania*. The combination of host and parasite factors will dictate how the disease develops.

*Leishmania* parasites have a wide range of adaptive mechanisms that disrupt several macrophage functions by altering host signaling pathways [8]. According to studies conducted on mice, the host endosomal Toll-like receptor-3 (TLR-3) recognizes the dsRNA genome of *Leishmania* RNA virus 1 (LRV1), causing tumor necrosis factor-α (TNF-α) expression and a strong type I interferon (IFN-I) antiviral immune response. This results in IL-6-driven hyperinflammation, an aggravation of the disease, and IL-17-dependent metastasis in IFN-γ-deficient (*Ifng*^−/−^) mice. These findings are consistent with patients with MCL *L. guyanensis* infection exhibiting high levels of IL-17 but low levels of IFN-γ [9,10,11]. The *Leishmania* parasite enters the mammalian host through blood-feeding from infected sand flies, where it activates the phagocytes, primarily macrophages [12]. *Leishmania* spp. can predominantly affect the skin and/or mucous tissue, depending on the infectious species. This can be considered as one of the factors influencing the type of cutaneous consequence and clinical implications, as well as influencing the immune system’s inflammatory and anti-inflammatory responses [13]. Macrophage cells swiftly initiate oxidative stress responses to eradicate pathogens and initiate stress-related signaling cascades that control inflammation and stress in the host defense against intracellular parasite infections [14,15].

When the immune system is regulating infectious diseases, Nrf2 (nuclear factor erythroid 2-related factor 2) is the main player due to its promotion of a pro-inflammatory and antioxidant profile balance. Over the course of evolution, these parasites have acquired methods for inducing this anti-oxidative pathway, which is promoted by Nrf2 signaling and results in a reduction in oxidative burst in the cell host. Through their evolutionary history, these parasites have developed strategies for activating this Nrf2-stimulated anti-oxidative pathway, which lowers the oxidative burst in the cell host. Nrf2 (nuclear factor erythroid 2-related factor 2), a transcriptional factor, has a role in controlling the oxidative stress response [16]. In fact, Nrf2 regulates the expression of several genes related to phase II enzymes and antioxidants [17]. Furthermore, Nrf2 activation suppresses inflammation via nuclear factor κB (NF-κB)-based dependent and independent pathways [18,19]. A negative regulator of NRF2, Kelch-like ECH-associated protein (KEAP1), is another important component in the inflammatory cascade. When KEAP1 binds to NRF2 in the cytosol, NRF2 is degraded under hemostasis circumstances [20]. Under oxidative stress, free NRF2 is translocated to the nucleus and complexes with ARE (antioxidant-responsive element) and MAF proteins due to changes in cysteine residues in KEAP1 [21]. Next, this complex attaches itself to the promoter region of antioxidant genes that code for cytoprotective antioxidant enzymes, including glutathione reductase (*Gsr*), heme oxygenase 1 (*HO-1*), NAD (P), and H Quinone Dehydrogenase 1 (*NQO1*) [22]. In order to maintain intracellular redox balance and regulate inflammation, Nrf2 and the downstream antioxidant genes it codes for are critical. More than 200 detoxifying and cytoprotective genes were discovered to be regulated by Nrf2 in response to oxidative stress [23,24,25]. Pathogens may trigger this network by a variety of methods, including the engagement of Toll-like receptors or the initiation of the PI3K/Akt pathway or endoplasmic reticulum stress [26]. Thus, it appears that the pathophysiological involvement of Nrf2 in parasite infections is just now coming to light. In this work, we discussed the functions of Nrf2 pathway activation or inhibition in leishmaniasis, as well as the prospective therapeutic applications.

## 2. Role of Nrf2 Signaling Pathway in Leishmaniasis

**Figure 1:**
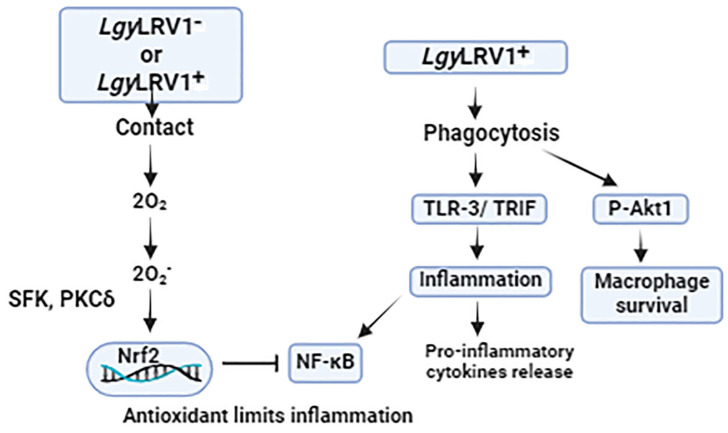
Schematic representation of mechanisms involved in *Leishmania* infection. Regardless of LRV1’s existence, the interaction between the parasite and the macrophage releases oxygen species produced by NOX2, which activates the Nrf2 pathway. This allows Nrf2 to be released from its negative regulator, KEAP1, and phosphorylated via SFK and PKC. The NF-κB inflammatory pathway and the synthesis of inflammatory chemokines and cytokines are restrained by this antioxidant response. *Leishmania* parasites that carry LRV1 improve the survival rate of infected macrophages and stimulate the production of Type-I interferon, inflammatory chemokines, and cytokines. This accelerates the spread of the infection through the release of IL-17.

Although KEAP1 primarily regulates Nrf2 stability, other activator proteins, such as iNOS, which is crucial for defending the host against *Leishmania*, and pathways involving TLR agonists, c-SRC, NADPH oxidase, and protein kinase C δ (PKCδ), which is controlled by phosphorylation by a non-receptor tyrosine protein kinase SRC (SRC), may also be involved in Nrf2 activation (Figure 1) [27,28,29]. PKCδ may then be involved in the phosphorylation of Nrf2 on serine 40, which causes Nrf2 to nuclear translocate and activate *Hmox1* transcriptionally in response to an oxidative stimulation [30]. With the exception of *L. amazonensis* infection, the underlying signaling pathway leading to Nrf2 activation in *L.* spp. infection is still poorly defined despite the fact that the increased expression of Nrf2-regulated genes like *Hmox1* suggests that Nrf2 is likely activated following infection with *L. donovani*, *L. chagasi*, or *L. braziliensis* [31,32,33]. In the latter instance, the PI3K/AKT pathway and dsRNA-induced kinase PKR are necessary for the Nrf2 signaling pathway [34]. Additionally, the phosphorylation of PERK can also cause Nrf2 activation in *L. amazonensis* infection, leading to an increased production of Nrf2-downstream genes such as *Hmox1*, which has anti-inflammatory and antioxidant effects [35]. There may be variations in Nrf2 activation amongst L. species. For instance, compared to *L. amazonensis* infection, more ROS are produced in *Leishmania* major infected cells, indicating that *L. amazonensis* may be able to more effectively control oxidative stress, maybe through Nrf2 [36]. Additionally, the way that different species kill intracellular *Leishmania* parasites varies. While some species suppress the production of ROS, *L. guyanensis*, for instance, is more vulnerable than *L. amazonensis* [37,38]. In this regard, a proteomic investigation reports that cells infected with *L. amazonensis* had a greater HO-1 level than cells infected with *L. major* [39]. As of now, NRF2 activation has only been linked to parasite survival and disease progression in cases of *L. amazonensis* infection [34].

*L. donovani*-infected macrophages showed differential modulation of around 10–16% of host mRNAs [40]. When *L. donovani* amastigotes or promastigotes infect macrophages, it is anticipated that certain upstream transcriptional regulators will undergo changes, including the suppression of STAT1. Additionally, this infection may be linked to the activation of specific mRNA subsets, such as Nrf2, IRF3, and IRF7, suggesting potential pathways for further investigation in understanding the immune response. Research shows that during *L. donovani* infection, the early stages of the parasite significantly drive transcriptional changes in macrophages. These alterations play an important role in shaping the host-cell responses, which can be both beneficial and detrimental. Understanding this dynamic could lead to more effective strategies for managing the infection. According to a different report, the regulation of inflammation and the redox balance of macrophages by the transcription factor Nrf2 are critical elements in the development of *L. infantum* infection [41]. They also emphasize the role of prostaglandin E2 (PGE2)/EP2r signaling in the maintenance of Nrf2 activation following infection, as well as the role of the NOX2/ROS axis in early Nrf2 activation.

One of the Nrf2 target genes that aids in heme metabolism and the synthesis of bilirubin, carbon monoxide, and free iron is *HO-1*. By inhibiting the cytokines generated during inflammatory processes, HO-1 increases *Leishmania* viability. Another target gene of Nrf2, which is increased under conditions of oxidative stress, is activating transcription factor 3 (ATF3). The adaptive response gene Atf3 is essential for both cellular processes and the transmission of signals from various receptors to either stimulate or suppress the expression of genes downstream. *Leishmania* survival depends on anti-inflammatory settings because Nrf2 upregulates the expression of *Atf3*. The function of *Atf3* in recruiting histone deacetylase 1 (HDAC1) during *Leishmania* infection was confirmed to be the cause of the epigenetic control of IL-12 and TNF-α [42,43]. Furthermore, their findings suggested that trigonelline hydrochloride, an NRF2 inhibitor, would be able to treat visceral leishmaniasis in mice infected with *L. donovani* [43,44]. Bichiou et al. investigated the influence of the parasite on the transcription of NRF2 and its target genes in bone-marrow-derived macrophages (BMdMs) generated from *Leishmania*-resistant and *Leishmania*-susceptible mice and suggested that *Leishmania* parasites enhanced the expressions of Nrf2, HO-1, Slc7a11, glutathione reductase (Gsr), CD36, and CAT [45]. They demonstrated that wortmannin administration decreased HO-1 protein expression and the phosphorylation of protein kinase B (PKB, or AKT), indicating the role of PI3K/Akt activity in the upregulation of HO-1 production during *Leishmania* infection [45]. Another study revealed *L. amazonensis* infection triggered the PERK/eIF2α/ATF4 signaling pathway in human tissue and macrophage cultures. Additionally, they showed that the infection with *L. braziliensis* increased the expression of HO-1 and Atf4 [46]. Research has demonstrated that GSK3 phosphorylates the Nh6 domain of NRF2, resulting in the proteasomal degradation of Nrf2 when infected with *L. amazonensis* [47]. Furthermore, it was demonstrated by Vivarini et al. that macrophages lacking NRF2 or PKR/Akt were able to upregulate ROS/RNS, downregulate the Nrf2-dependent gene Sod1, and exhibit a reduced parasite burden [34]. Additionally, *L. amazonensis* inhibited Keap1 by upregulating *p62* via *PKR* [34].

**Figure 2:**
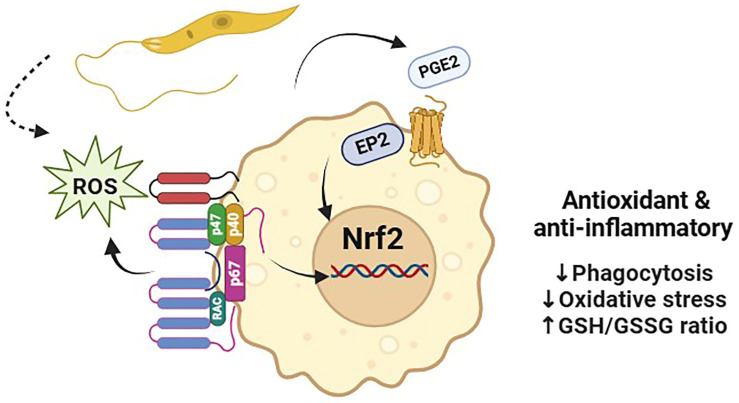
*Leishmania* infection is significantly influenced by the Nrf2, which regulates macrophage redox balance and inflammation. *Leishmania* infection mediates the activation of NADPH oxidase, which releases Nrf2 from its negative regulator KEAP1, allowing Nrf2 to translocate into the nucleus. Nrf2 nuclear translocation is significantly reduced if PGE2 synthase and the EP2 receptor are pharmacologically blocked in the latter stages of infection.

Nrf2 expression during *Leishmania* infections was found to be reliant on NADPH oxidase 2 and the SRC family of protein tyrosine kinase (SFK) signaling, which resulted in its translocation into the nucleus and the activation of particular downstream genes [48,49]. Additionally, they observed that *Leishmania*’s interaction with the cell surface and phagocytosis was critical in reprogramming host-cell metabolism in a way that was dependent on Nrf2. Furthermore, Nrf2 restricted inflammation and pathology by regulating the levels of the anti-*Leishmania* cytokine TNF-α, which could lead to tissue destruction in patients with mucocutaneous leishmaniasis. *L. guyanensis* parasites are known to carry an endosymbiotic dsRNA virus that exacerbates the disease and spreads the infection (Figure 2).

In macrophages infected with *L. major* and *L. amazonensis,* the LC3-II/Act ratio was observed to be upregulated, whereas NO generation was found to be decreased after 24 h of infection [50]. According to another study, autophagy activation raises the intracellular load of *L. amazonensis* in macrophages [51]. Furthermore, because PKR phosphorylates eIF2a, which is essential for controlling the production of autophagosomes, PKR-deficient cells exhibit decreased autophagic processes [52]. *L. amazonensis*-infected macrophages exhibit decreased levels of Keap1, which allows Nrf2 to translocate into the nucleus and alter the expression of ARE-dependent genes [53]. According to Dias-Teixeira et al. [35], *L. amazonensis* infection also resulted in decreased Nrf2 expression, nuclear translocation, reduced HO-1 expression, and high NO generation in ATF4 (activating transcription factor 4)-knockdown macrophages. According to the same study, endoplasmic reticulum stress-induced phosphorylation of PERK resulted in Nrf2 activation, which in turn promoted *L. amazonensis* infection by increasing the Nrf2/ATF4 regulation of ARE in the HO-1 gene promoter and causing ATF4 dimerization in the nucleus. The phlebotomine sandfly *Lutzomyia longipalpis* Saliva stimulates Nrf2 and the HO-1 target gene to be expressed by macrophages in situ and in human skin at the bite site, demonstrating the mechanism by which sandfly-borne vectors transfer and establish *Leishmania* infections [54].

Notwithstanding their commonalities, the *Leishmania* species exhibit distinct virulence and pathogenicity patterns based on the host’s immunological makeup. According to de Menezes et al. [39], a proteomics analysis comparing the infections of *L. major* and *L. amazonensis* revealed that the latter does not use the Nrf2 pathway’s canonical signature to try to subvert host-cell defenses. This was demonstrated by a significant increase in the expression of SQSTM1 (p62) and HO-1 in *L. amazonensis*-infected macrophages. Moreover, macrophages infected with *L. donovani* take advantage of Nrf2 activation. With the goal of surviving inside macrophages, these parasites use Nrf2’s direct binding to the Tollip promoter to enhance the production of Tollip (Toll-interacting protein), a negative regulator of the IL-1R/TLR pathway’s activation [31].

## 3. Pharmacotherapy of Leishmaniasis via Nrf2

Some scientists have explored exogenous drugs or plant-derived extracts that may target Nrf2 or ARE-responsive genes in an effort to find potential options for treating leishmaniasis. According to Cataneo et al., quercetin reduced *L. braziliensis* promastigotes’ survivability rates by the Nrf2 signaling pathway [55]. Recent research indicates that dehydroabietic acid (DHA) may have anti-protozoan properties against *L. braziliensis*, *L. infantum*, and *L. donovani* [56]. According to Goncalves et al., DHA inhibited the growth of promastigotes of *L. amazonensis* by downregulating the expression of NRF2/ferritin, increasing the amount of free iron and iron bound to transferrin, and producing ROS [57]. In addition to modulating Nrf2 in leishmaniasis, these compounds also directly promote an apoptosis-like process in the promastigote and amastigote forms of *L. amazonensis*, which results in a decrease in ROS, nitric oxide, TGF-β, and IL-10, followed by an increase in Nrf2/HO-1/Ferritin expression, which modulates the parasites’ intracellular proliferation [56,58].

According to Tomiotto-Pellissier et al. [59], extracts from the Brazilian Cerrado plant *Caryocar coriaceum* have a leishmanicidal effect on the amastigotes and promastigotes forms of *L. amazonensis* by upregulating Nrf2/HO-1/Ferritin expression. This lowers the labile iron pool in infected macrophages, which in turn lowers the parasite’s rate of replication.

According to Chowdhury et al. [60], a new ABC transporter, identified as ABCC2 or *L. donovani* multidrug resistance protein 2 (LdMRP2), is overexpressed in baicalein (BLN)-resistant parasites (pB25R). Macrophage MRP2 transporter overexpression is accompanied by amastigote resistance. Moreover, the PI3K-mediated Nrf2 translocation was found to trigger MRP2 expression in macrophages during infection. According to Das et al. [32], miltefosine treatment reversed the HO-1 level and Nrf2 activation, thereby reducing the HO-1/ERK/Nrf2-dependent *L. donovani* load.

## 4. Discussion

Extensive findings suggest that Nrf2-driven anti-oxidant mechanism in leishmaniasis is mediated by central signal transducers, including PKR, PERK, and PI3K/Akt. Furthermore, it has not yet been shown that the components of the Nrf2 pathway have undergone post-translational changes. It is unknown if these kinases, other than GSK3, phosphorylate this transcription factor directly or through intermediary molecules that lead to its activation. To continue with the gaps, more study is needed to determine which macrophage receptors are responsible for the pathway’s activation as well as which parasite antigens bind to these receptors. There exist variations in the way that distinct *Leishmania* species activate Nrf2. The activation of nuclear translocation and Nrf2 activity by *L. donovani* infection also reduces oxidative stress; however, the molecular partners needed to initiate this signaling remain unknown. It is known that Nrf2 expression and activation happen during the first interaction between the cell host and the parasite; this increases the quantity of gene products linked to an antioxidant profile and M2 macrophage properties like anti-inflammatory spectrum; additionally, Nrf2 knockout cells or their inhibition reduce the parasite infection.

Certain plant extracts and purified compounds have the ability to alter the Nrf2 pathway in an unclear fashion, either with a leishmanicidal impact or by activating it through an as-yet-unidentified mechanism. The first oral treatment for leishmaniasis, miltefosine, offers significant promise as it may block some target genes and components of the Nrf2 pathway, resulting in a shift in the profile of macrophages that is proinflammatory [61]. However, what intracellular targets do these products have?

The recruitment and infiltration of monocyte cells and NK cells at the site of inflammation are influenced by Nrf2 activation. This process is facilitated by Interleukin (IL)-17D, which is a target of Nrf2 [62]. What level of Nrf2 modulation would be required to maintain the homeostasis of both host and parasite cells without causing harm to each cell’s secondary functions? Targeting the Nrf2 pathway may have unfavorable effects on the effectiveness of the interaction between the parasite and the host cell. More research is necessary to uncover the main sensors implicated in Nrf2 activation by different *Leishmania* species. Finding Nrf2’s molecular interactions and the variety of genes that could be involved in oxidative burst will provide new opportunities for choosing potential treatment targets.

## 5. Conclusions

It is indisputable that Nrf2 coordinates cellular function. Classifying a component such as NRF2 as having a positive or negative influence during the course of a parasitic infection is challenging because of the molecular complexity that separates parasite–host cell interactions. Overall, NRF2 has a dual role in parasitic infection that can be advantageous to the parasite and the host by triggering the antioxidant and anti-inflammatory response. Since oxidative stress is a component of the host–parasite interaction in infectious diseases like leishmaniasis, the inhibition of Nrf2 may worsen the condition or have unintended consequences that need to be investigated. Therefore, while developing innovative therapeutic strategies for *Leishmania* infections, it may be crucial to look into how different signaling pathways might either activate or inhibit NRF2. Ultimately, further research is needed to determine the critical sensors that different pathogen species use to activate Nrf2.

## Data Availability

Not applicable.
